# A workflow to generate patient-specific three-dimensional augmented reality models from medical imaging data and example applications in urologic oncology

**DOI:** 10.1186/s41205-021-00125-5

**Published:** 2021-10-28

**Authors:** Nicole Wake, Andrew B. Rosenkrantz, William C. Huang, James S. Wysock, Samir S. Taneja, Daniel K. Sodickson, Hersh Chandarana

**Affiliations:** 1 Department of Radiology, Montefiore Medical Center, Albert Einstein College of Medicine, 111 East 210th Street, Bronx, NY 10467 USA; 2 Center for Advanced Imaging Innovation and Research (CAI2R) and Bernard and Irene Schwartz Center for Biomedical Imaging, Department of Radiology, NYU Langone Health, NYU Grossman School of Medicine, New York, NY USA; 3 Department of Urology, NYU Langone Health, NYU Grossman School of Medicine, New York, NY USA

**Keywords:** Augmented reality, Three-dimensional, Medical imaging, Urologic oncology, Surgical planning

## Abstract

Augmented reality (AR) and virtual reality (VR) are burgeoning technologies that have the potential to greatly enhance patient care. Visualizing patient-specific three-dimensional (3D) imaging data in these enhanced virtual environments may improve surgeons’ understanding of anatomy and surgical pathology, thereby allowing for improved surgical planning, superior intra-operative guidance, and ultimately improved patient care. It is important that radiologists are familiar with these technologies, especially since the number of institutions utilizing VR and AR is increasing. This article gives an overview of AR and VR and describes the workflow required to create anatomical 3D models for use in AR using the Microsoft HoloLens device. Case examples in urologic oncology (prostate cancer and renal cancer) are provided which depict how AR has been used to guide surgery at our institution.

## Introduction

In modern surgery, pre-operative imaging techniques such as computed tomography (CT), magnetic resonance imaging (MRI), and ultrasonography are used for both pre-surgical planning and intra-operative guidance. Although these imaging techniques allow surgeons to visualize the surgical anatomy, they are limited since interpretation of the three-dimensional (3D) anatomy must be made on a two-dimensional (2D) screen. Approximately 30 years ago, in the late 1980s and early 1990s, academic radiologists attempted to improve radiological image visualization by using enhanced image post-processing methods such as volume rendering and maximum intensity projection (MIP) techniques [1–4] and the radiology “3D imaging laboratory” emerged.

However, while the methods of image visualization used in these laboratories allow for improved understanding of anatomy as compared to viewing images in 2D, they are still limited by the fact that 3D data is being visualized on a 2D screen. Specifically, volume rendering techniques provide a 2D representation of a 3D volume and can define certain complex anatomy, but these methods are limited by overlapping structures [5]. In addition, volume rendering is vulnerable to interobserver variability because the user must adjust the display parameters (i.e. the level of opacity) [6]. MIP techniques are valuable for the evaluation and display of the vasculature in CT angiography, however high-attenuation voxels may obscure the vasculature so that the 3D relationship among the structures cannot be visualized [5].

Recently, due to advancements in computer power and manufacturing technology, VR and AR technologies have become widely available, and they are being utilized directly at the point of care. It is believed that these methods of advanced image visualization can improve understanding of anatomy and surgical pathology, thereby enhancing surgical planning, providing improved intra-operative guidance, and ultimately improving patient care.

VR is a completely immersive computer-generated simulation of a 3D environment that can be interacted with using hand controllers. VR has been around for over 50 years, with the first head-mounted display, the Telesphere Mask, patented in 1960 [7]. The Telesphere Mask provided stereoscopic 3D television, wide vision, and true stereo-sound. Current VR head mounted displays provide separate images for each eye, stereo sound, head motion tracking sensors, and gaming controllers. Some examples of VR headset devices include the HTC Vive and Valve, Oculus Rift, Oculus Quest, Oculus Quest 2, Samsung Gear VR, and Playstation VR. Most of these headsets are tethered and must be run through a computer with a powerful graphics processing unit (GPU). The Oculus Quest and Quest 2 were designed specifically for untethered experiences at 90 Hz with software such as Virtual Desktop (vrdesktop.net) and Air Link (Oculus, Facebook Technologies, LLC, Menlo Park, CA). Finally, VR viewers such as Google’s Cardboard have been designed for smartphones. For this type of VR, rather than using internal dedicated displays, the VR content is viewed on the smartphone screen and the lenses in the viewer act as a stereoscope.

**Fig. 1:**
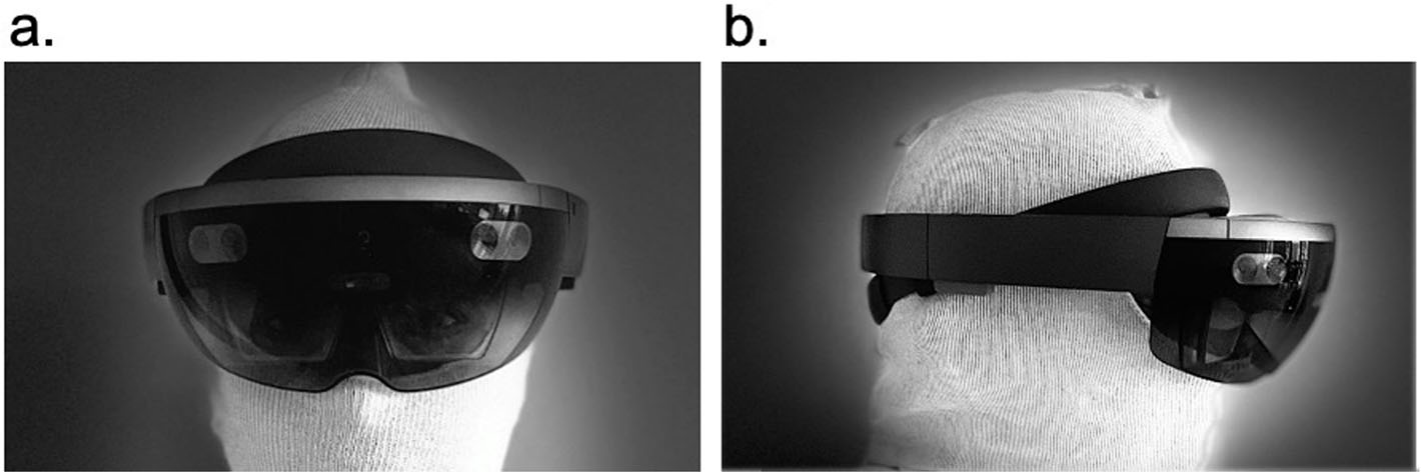
**a** Front and **b**) side views of the Microsoft HoloLens AR Headset

Unlike VR which is completely immersive, AR is a technology that allows 3D computer graphics to be placed in the real environment in the form of an overlay. In the 1960’s Ivan Sutherland created the first AR head mounted display system with the help of his student Bob Sproull [8]. This head mounted display is referred to as the “Sword of Damocles” because it was so heavy that it had to be suspended from the ceiling above the user’s head. Some examples of AR headsets or glasses include the Microsoft HoloLens 1 (Fig. 1**)**, Microsoft HoloLens 2, Magic Leap 1, Epson Moverio BT-300FVP, Google Glass Enterprise Edition, Vuzix Blade AR, Meta 2, Optinvent Ora-2, Garmin Varia Vision, and HP Reverb G2. AR can also be experienced with smartphone driven platforms such as Apple’s ARKit and Google’s ARCore, which superimpose digital objects into the real world on a screen. Here, instead of wearing a headset or glasses, virtual 3D objects are viewed on a smartphone device that visualizes the real surrounding environment through its camera.

**Fig. 2:**
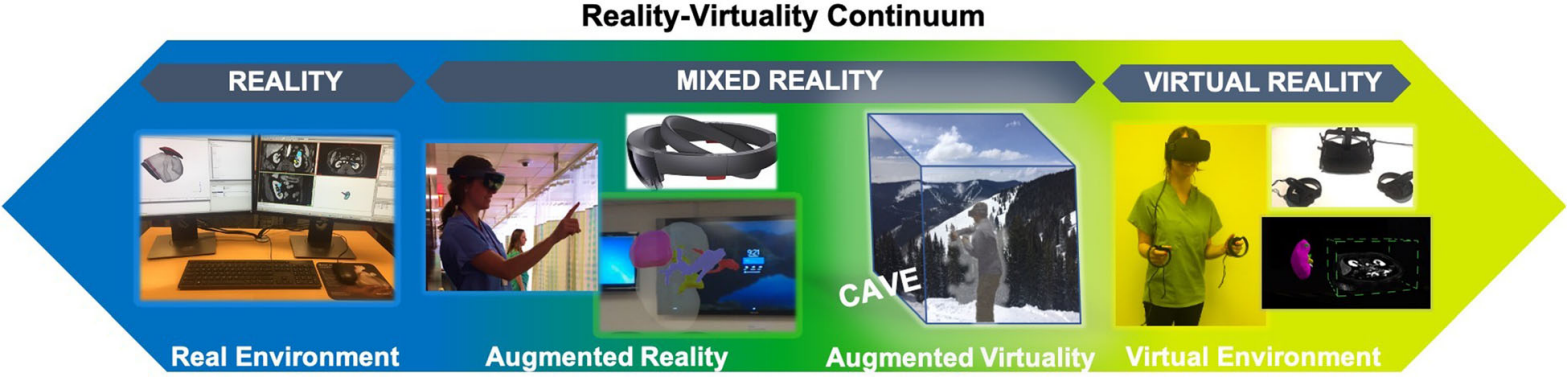
Taxonomy of reality environments (Adapted from Milgram and Kishano’s reality continuum)

A taxonomy of reality environments was first proposed by Milgram and Kishano in 1994 [9]. In this taxonomy, different types of reality are described on a continuum with the real environment on one end of the continuum and the virtual environment on the other end **(**Fig. 2**)**. On this continuum, AR and augmented virtuality are categorized as mixed reality, with augmented virtuality referring to virtual spaces where physical elements (i.e. people or physical objects) are dynamically integrated into the virtual world in real-time. Augmented virtuality is more commonly referred to as a CAVE system which stands for Cave Automatic Virtual Environment where projectors are directed towards the walls in a room. Today, the term “mixed reality” is sometimes used interchangeably with AR. It is important to note that most of Windows Mixed Reality is essentially VR that is compatible with Windows 10 computers. Examples of Microsoft compatible mixed reality headsets that are currently available include the Samsung HMD Odyssey, Acer Headset, Dell Visor, HP Headset, and Lenovo Explorer.

There are many applications of both VR and AR in medicine including pre-operative planning [10, 11], surgical simulations [12, 13], intra-operative guidance [11, 14–16], surgical navigation [17–19], and trainee education [13, 20]. Although AR and VR have been gaining momentum over the past few years, experiences using VR/AR technologies in medicine remain fairly sparse and the role of AR in medicine is yet to be defined. In the field of urologic oncology, a recent review on 3D printing and AR/VR technologies found 44 publications with 29 on renal cancer, 13 on prostate cancer, and 2 describing both kidney and prostate cancer [21].

**Fig. 3:**
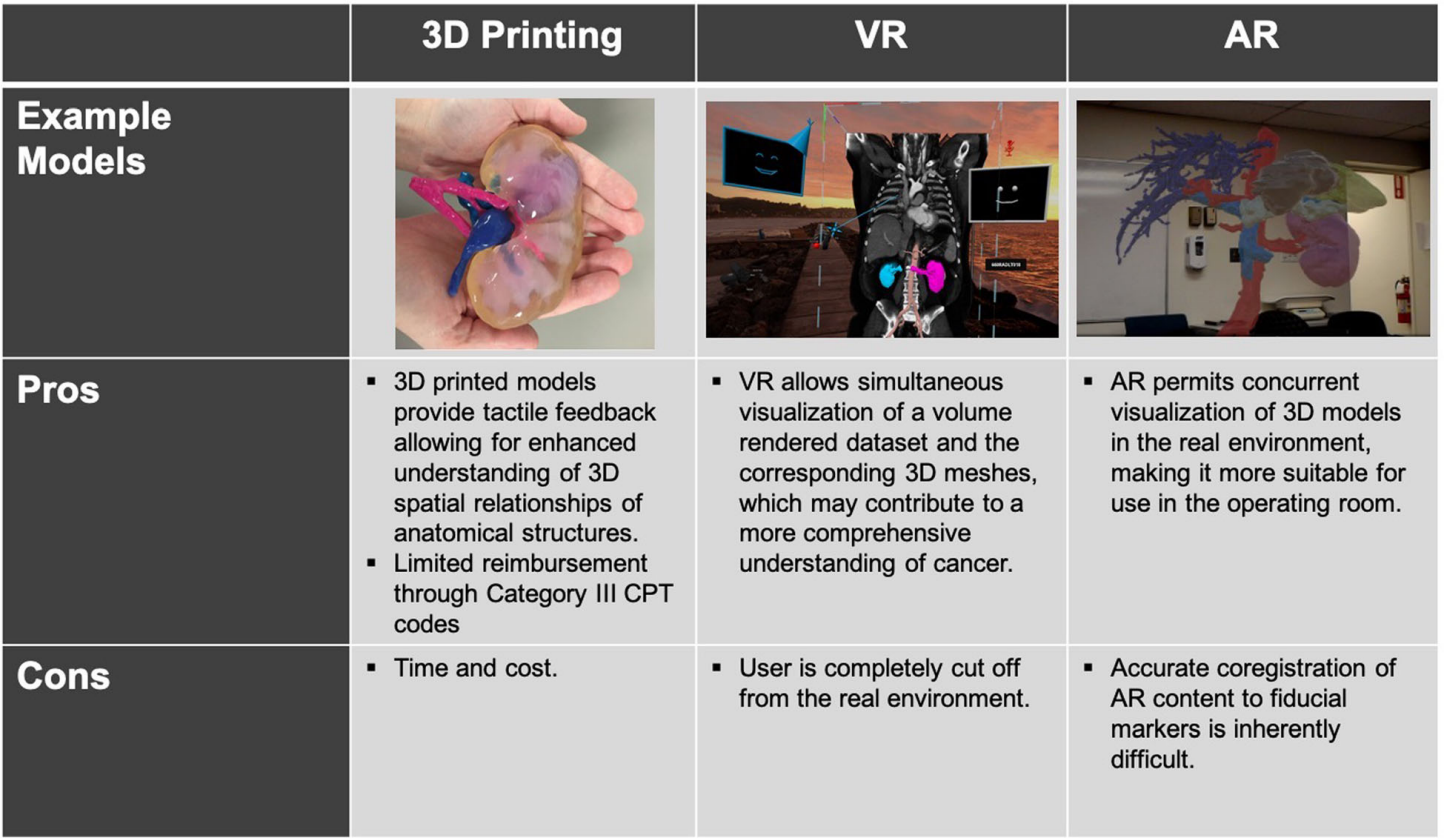
Pros and Cons of 3D Printing, VR, and AR. A multi-colored 3D printed renal cancer model printed with material jetting (Connex 5, Stratasys, Eden Prairie, MN) is shown on the left. Middle: VR visualization of a sagittal CT scan with segmentation of the kidneys (syGlass, Morgantown, WV) viewed through an Oculus Rift headset. Right: AR visualization through the Microsoft HoloLens Device

Each of these methods of advanced visualization has its pros and cons **(**Fig. 3**).** What role these technologies will play in clinical practice will be determined as more institutions implement these methods of advanced visualization and clinical studies are performed evaluating the uses and impact that these methods on patient care.

In this article, we report our experience creating AR models to facilitate planning urologic surgical procedures with the aim of teaching clinicians about AR technology and how it can be implemented in clinical practice. Specifically, we describe our laboratory’s AR modeling workflow and demonstrate this through illustrative case descriptions for kidney and prostate cancer.

## Workflow to create patient-specific AR models

**Fig. 4:**
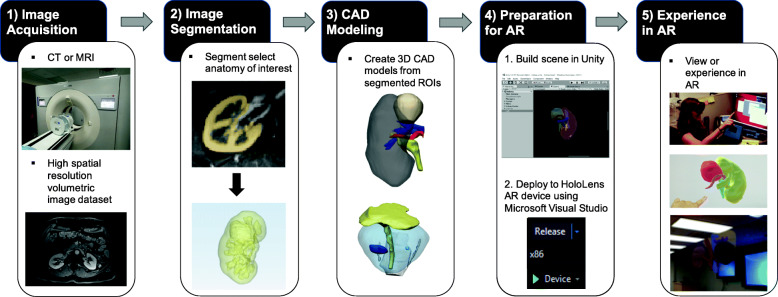
AR Workflow including the following steps: 1) Image Acquisition, 2) Image Segmentation, 3) CAD Modeling, 4) Preparation for AR, and 5) Experiencing in AR

AR devices do not currently accept Digital Imaging and Communications in Medicine (DICOM) images as an input. Typically, the entire workflow consists of 1) medical image acquisition, 2) image segmentation to delineate the appropriate anatomical ROIs to be included in the AR visualization, 3) computer-aided design (CAD) Modeling, 4) Preparation for AR, and 5) experiencing in AR. Our institution’s approach for creating patient-specific AR cancer models is shown in Fig. 4 and described below.

### Image acquisition

Image acquisition may be tailored to ensure that the appropriate anatomic structures are well-visualized which can greatly simplify the workflow to create patient-specific models for AR and ensure the creation of high-quality content. For all image acquisitions, pertinent factors include the following: volumetric data acquisition, high spatial-resolution, small slice thicknesses, high signal-to-noise and contrast-to-noise ratios, and minimal image artifact [22].

CT is the most common clinical technique adopted to generate 3D anatomical models or AR visualization due to the relative ease of post-processing CT data [23]. CT provides information about tissue density and contrast enhancement and thus has relatively simple signal content. The signal intensity is proportional to the tissue density and the Hounsfield Unit (HU) scale is a linear transformation of the original linear attenuation coefficient measurement. Over a large range of acquisition parameters, specific tissues will have the same (or very similar) Hounsfield units. Compared to CT datasets, MRI data offers advantages since it provides exquisite soft tissue contrast needed for accurate segmentation without exposing patients to unnecessary ionizing radiation. MRI, however, often requires complicated imaging techniques in order to generate high resolution 3D anatomic models [24]. Our image acquisition approaches for renal and prostate cancer are described below.

#### Renal Cancer

Multi-phase high resolution CT or MRI may be performed. For MRI, image acquisition can be performed on a 1.5 T or 3 T system. We routinely perform imaging on a 1.5 T system and prefer to use contrast-enhanced 3D T1-weighted acquisition as this provides detail contrast information as well as relatively high spatial resolution. A 3D post-contrast fat-suppressed gradient-echo T1-weighted sequence with an interpolated spatial resolution of 1.4 mm × 1.4 mm × 2 mm is used to generate the 3D renal models. Standard sequence parameters are: TR = 3.58 ms, TE = 1.3 ms, FA = 12°, acquisition time of breath-hold ranging from 13 to 20 s.

#### Prostate Cancer

Multi-parametric MRI on a 3 T magnet with a pelvic phased-array coil is performed (Siemens, Erlangen, Germany) to generate prostate cancer models. The acquisitions include multi-planar T2-weighted images (T2WI), diffusion-weighted images (DWI) with a calculated b-1500 s/mm^2^ image set, and dynamic contrast enhanced (DCE) images. Standard 2D TSE axial T2WI images are acquired with a 0.7 × 0.7 × 3.0 mm^3^ resolution and 3D T2WI Sampling Perfection with Application optimized Contrasts using different flip angle Evolutions (SPACE) with a 0.6 × 0.6 × 1.0 mm^3^ resolution. In order to achieve optimal visualization of prostate neoplasms with high contrast and spatial resolution we also utilize DWI, although DWI sequences tend to have a lower spatial resolution than other anatomic sequences. The spatial resolution for DWI at our institution is 1 × 1 × 3 mm^3^.

### Image segmentation

Image segmentation is one of the most important tasks in generating accurate patient-specific 3D anatomical models and is often the first and most critical step in many clinical applications. The goal of image segmentation is to partition a volumetric medical image into separate regions, usually organs or diseased structures. Segmentation algorithms for gray-scale images are generally based on one of two basic categories dealing with properties of intensity values: discontinuity (partitioning an image based on abrupt changes in gray level) and similarity (partitioning an image into regions that are similar).

Common image segmentation techniques include thresholding, edge detection, and region growing. Image thresholding, the simplest method of image segmentation, is used to partition an image into a binary image representing the segmentation. To differentiate pixels of interest, a comparison of each pixel intensity value with respect to a threshold is performed. If the pixel’s intensity is higher than the threshold, the pixel is set to one value, i.e. white, and if it is lower than the threshold then it is set a second value, i.e. black. In more sophisticated implementations, more than one threshold may be specified, so that a band of intensity values can be set to one value while everything else is set to the other. This is a common method used for image segmentation to generate 3D models.

Edge pixels are pixels at which the intensity of an image function changes suddenly and the edges are sets of connected pixels. Edge pixels can be detected by local image processing methods called edge detectors. Edge detection is used most frequently for segmenting images based on sudden local changes in intensity. Two principal properties used for establishing similarity of edge pixels are 1) the strength of the response of the gradient operator used to produce the edge pixels and 2) the direction of the gradient. Edge detection can be made more selective by smoothing the image prior to computing the gradient.

Another technique, region growing, groups pixels or sub-regions into larger categories based on predefined growth criteria. The basic approach is to start with a set of “seed” points and from these grow regions by appending to each set of neighboring pixels that have predefined properties similar to the seed.

At our institution, in order to create 3D anatomical models for use in 3D printing, AR, and VR, image segmentation is first performed with Mimics software (Materialise, Leuven, BE). All digital imaging and communication in medicine (DICOM) images are imported to a dedicated Mimics workstation, which is used for 3D visualization and image segmentation. Each desired anatomical region of interest (ROI) is selected using a combination of tools including thresholding, region growing, and painting with multiple slice interpolation.

#### Renal Cancer

**Fig. 5:**
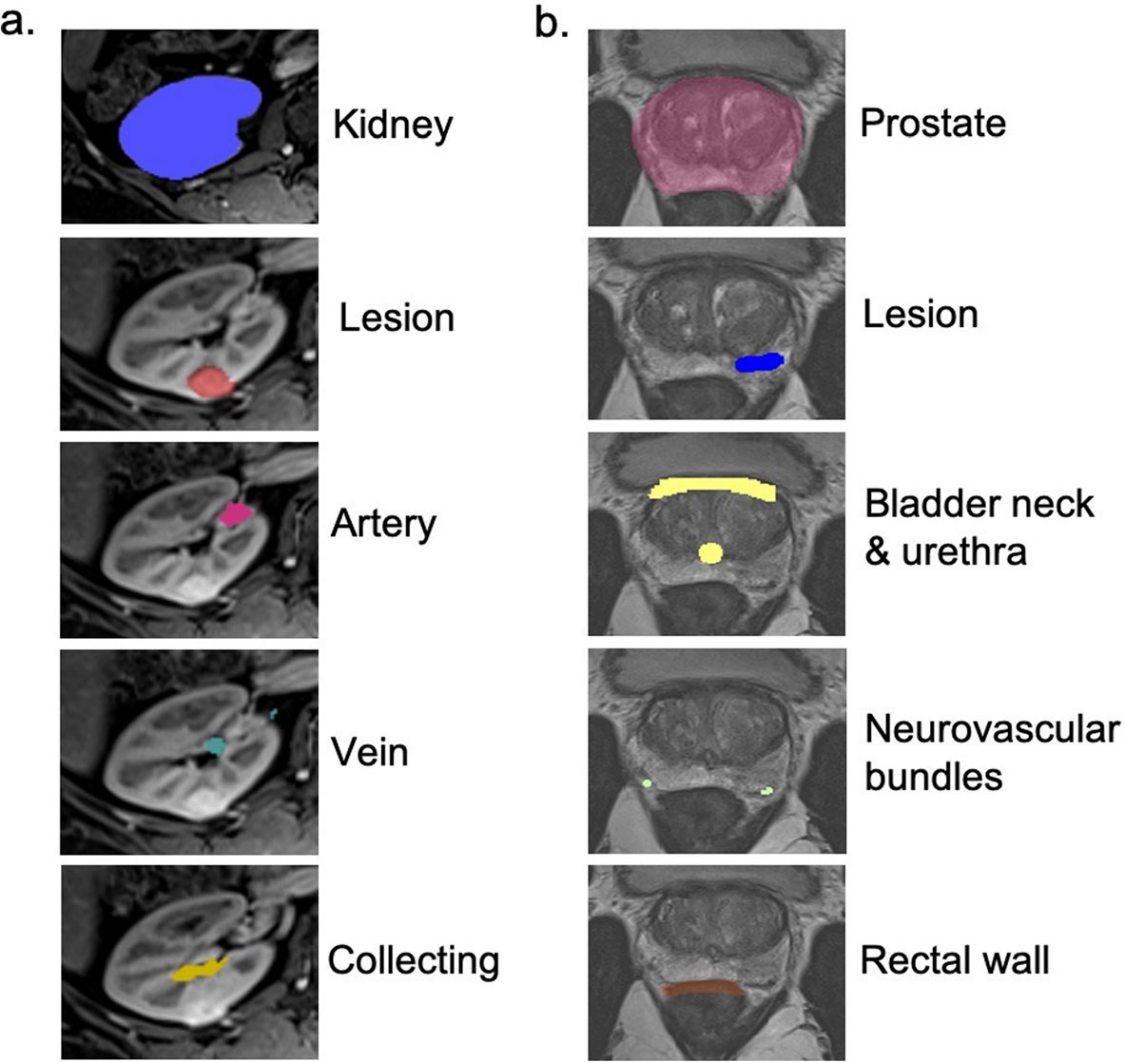
Image segmentation of anatomical ROIs for **a**) kidney cancer models and **b**) prostate cancer models

For renal cancer models, the kidney, tumor, renal artery, renal vein, and collecting system are segmented as five separate anatomical ROIs **(**Fig. 5a**)**. Post-contrast arterial, venous, and delayed phases are co-registered using a 3D rigid registration method that includes translation and rotation. Co-registered images are overlaid for the segmentation., The kidney is segmented from background and other organs by first using a threshold method. Next, the kidney is roughly painted every 10 slices on the axial images and filled in with the selected threshold using the multiple slice interpolation tool. The remaining structures are manually selected every few slices on the axial images and are filled in using the multiple slice editing tool. For this cohort of patients, the total time required for segmentation was approximately 2 h per case. Note that it is also possible to segment the kidney, tumor, and renal artery from the arterial phase images, renal vein from the venous images, and collecting system from the delayed phase images and then the segmented anatomical regions of interest can be brought together in CAD software [25].

#### Prostate Cancer

The prostate, suspicious focal lesion, prostatic urethra, urinary bladder, neurovascular bundles, and rectal wall are segmented. The lesion is usually segmented manually on the DWI dataset, while the remaining structures are segmented using the 3D SPACE images. The prostate is segmented using the 3D interpolate tool, where ROIs can be drawn on axial, sagittal, and coronal slices; once a few ROIs are outlined, then the program automatically interpolates and fills in the prostate. The rectum, urethra, and bladder are segmented by drawing ROIs every 5–10 slices on the axial images and using the multiple-slice interpolate tool **(**Fig. 5b**)**. Co-registration of the segmented lesion is performed using a 3D rigid registration method consisting of translation and rotation. Segmentation times range from 1 to 4 h depending on the difficulty of the case.

Although we have included segmentation times above, it is important to recognize that these will vary based on the image post-processing software that is utilized as well as the experience of the user.

### CAD Modeling 

**Fig. 6:**
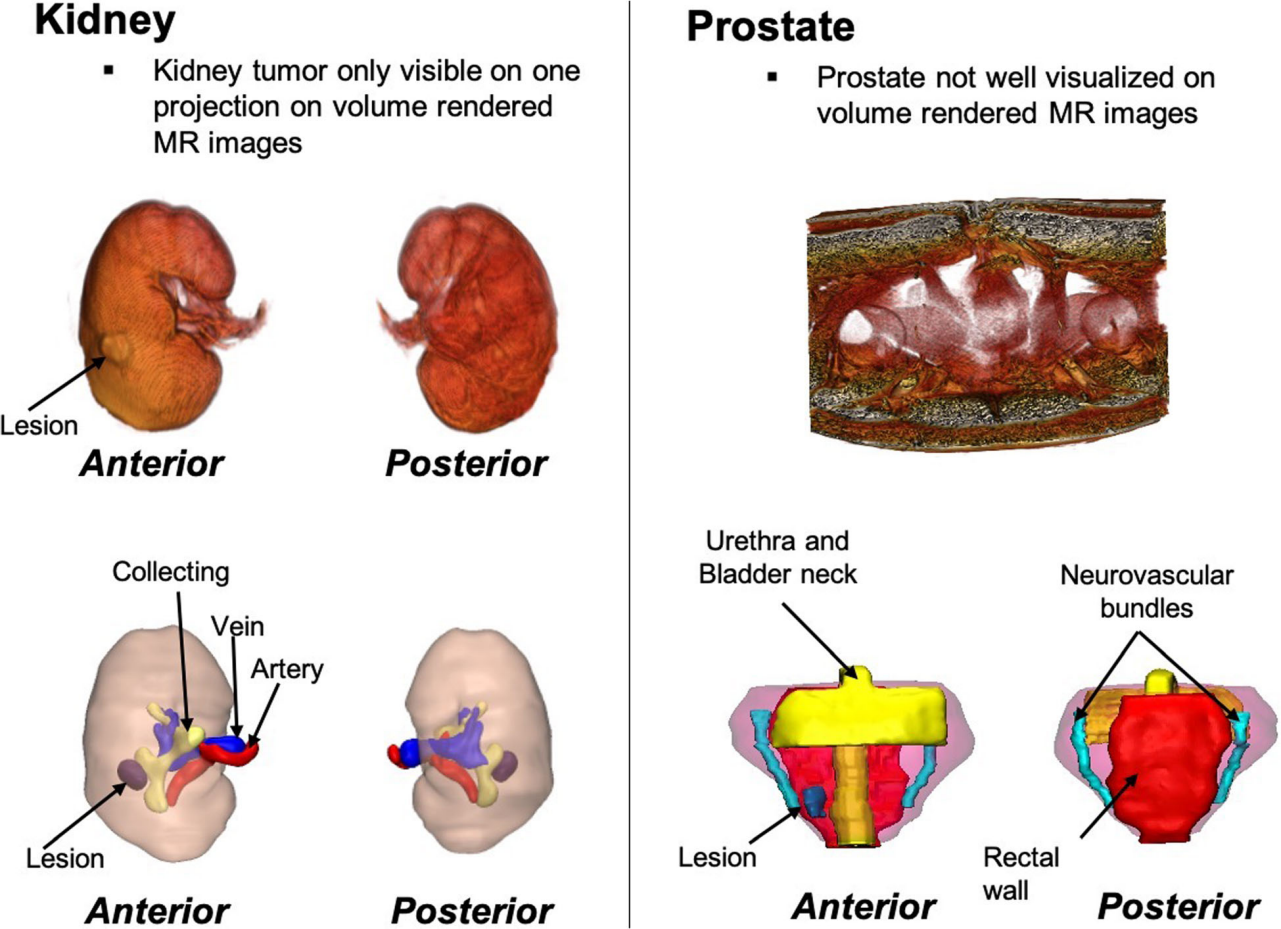
3D visualization of volume rendered images (top row) and 3D anatomy derived from image segmentation (bottom row). All pertinent anatomy could be appropriately segmented and visualized in 3D

After image segmentation, each ROI is converted to a 3D surface mesh which is exported in STL file format, the most common file format for 3D printing. Marginal local smoothing (smoothing diameter = 5–15 mm, smoothing strength = 1) is performed to minimize the pixelated appearance (3-matic, Materialise, Leuven, BE), contours are created and overlaid onto the DICOM images to ensure that the STLs accurately reflect the anatomy, and all anatomical structures are viewed simultaneously in 3D. The STL files are converted to OBJ format in order to be imported directly into the Unity game engine, version 5.4 (Unity Technologies, San Francisco, CA, USA). For abdominal and urological organs, where multiple structures have similar signal intensities, 3D representations of segmented anatomy are preferred over traditional volume renderings **(**Fig. 6**).**

Other post-processing software programs such as ZBrush, Blender, Freeform, MeshLab, and MeshMixer are available which can optimize surface meshes for 3D printing, AR, and VR. It is important to note that different degrees of wrapping and smoothing meshes in these programs may cause a loss of detail. In addition, it is essential to check for accuracy of both segmentation and post-processing as a last step before printing or utilizing these models in AR or VR.

### Preparation for AR

**Fig. 7:**
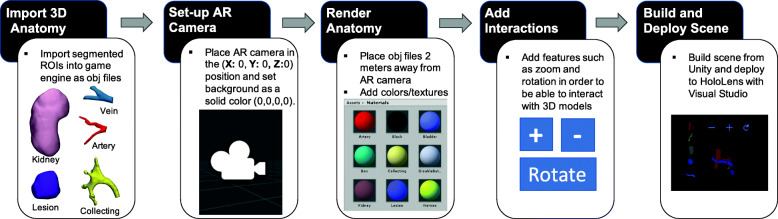
Overview of workflow to prepare 3D segmented anatomical ROIs for AR using Unity and Microsoft HoloLens

As described in the introduction, many AR devices are currently available, and it is expected that these technologies will continue to develop and gain popularity. In order to import 3D anatomic models into the AR device (HoloLens 1, Microsoft, Redmond, WA), a powerful game engine is utilized (Unity, Unity Technologies, San Francisco, CA). Our workflow to convert 3D segmented anatomic ROIs is shown in Fig. 7**.** First, OBJ files are imported into the software. Second, an AR camera is placed as (**X:** 0, **Y:** 0**, Z:**0) and the camera background is set to a solid color with the **RGBA** values set to (0,0,0,0). Next, each ROI is rendered approximately two meters away from the main camera (**X:** 0, **Y:** 0**, Z:** 2) and different colors are assigned to represent the anatomy of interest. In order to allow interaction with the 3D anatomy in AR, voice commands such as *show kidney* and actions such as *zoom* and *rotate* are added to each model. Finally, the scene is sent to the AR device using Microsoft Visual Studio (Microsoft, Redmond, WA). Note that a similar workflow could be applied for other types of AR or VR headsets.

### Experience in AR

**Fig. 8:**
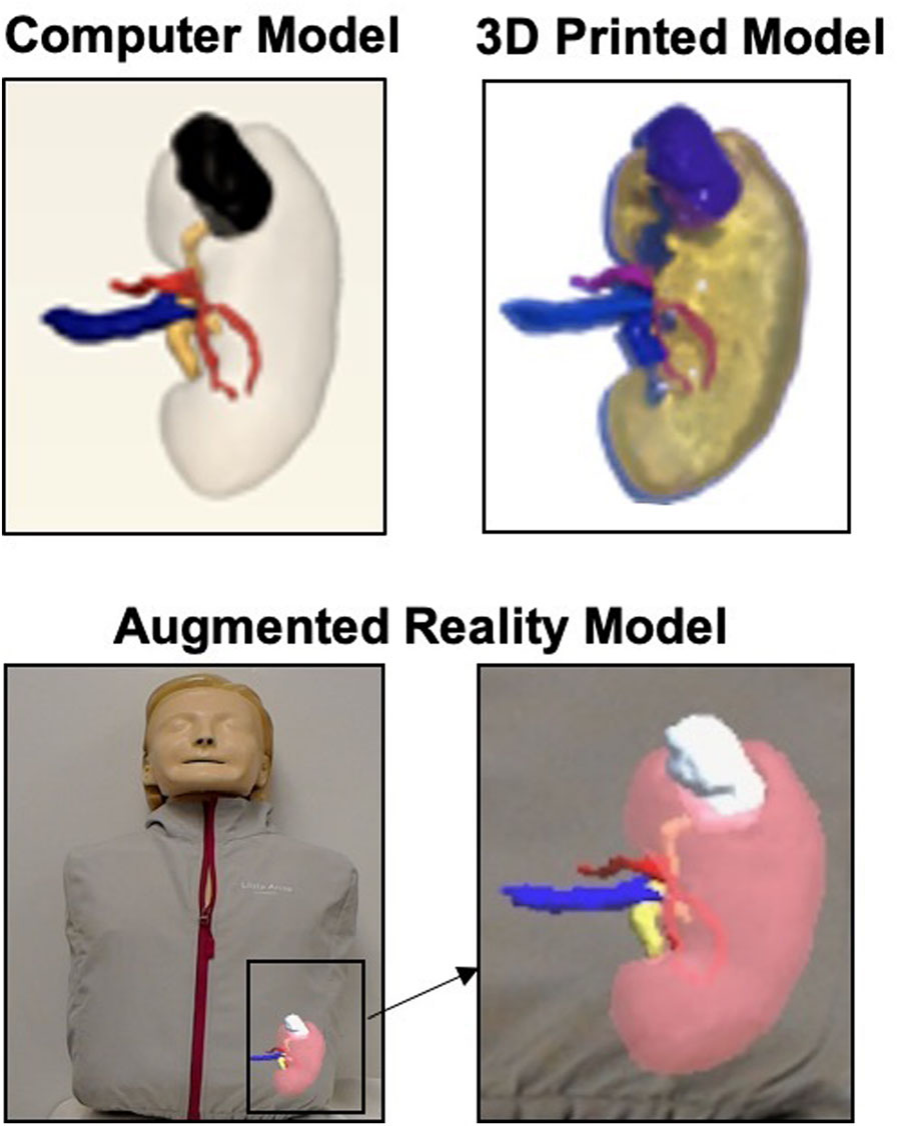
3D computer model, printed model, and AR model shown projected onto a mannequin using visual co-registration

Full color holograms are rendered in AR in the user's physical space with the AR device (HoloLens 1, Microsoft, Redmond, WA). To start an AR experience, an anatomical scene can be selected using the *air tap* hand gesture command or a voice command. To select using the *air tap* gesture, the hand is held up in front of the headset, the index finger and thumb are quickly pressed together, and released. In order to make a model bigger or smaller, the user can use the *air tap* gesture to click on a plus or minus button within the AR scene. Similarly, to rotate an object, the user can use the *air tap* gesture and hold the index finger and thumb together while moving the hand up, down, left, or right. Voice commands such as *zoom in*, *zoom out,* and *rotate* can also be used to perform these tasks. In this experience, using a subjective visual assessment of our AR models, we believe that they accurately depict the anatomy and closely resemble both computer and 3D printed models **(**Fig. 8**).**

## Case discussions

At our institution, we have an ongoing Institutional Review Board (IRB) approved prospective 3D model study evaluating the impact that 3D printed and AR models can make in patient care. These models are utilized by surgeons and shown to patients pre-operatively. If desired, surgeons could also use the AR models intra-operatively. Case discussions for some of these AR models are described below.

### Renal Cancer

**Fig. 9:**
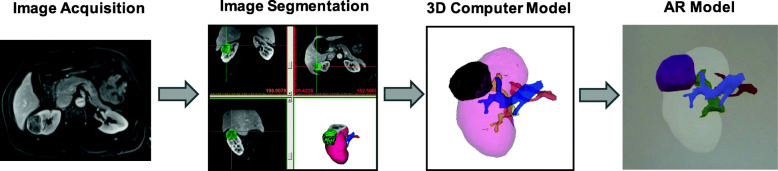
Image Acquisition**,** segmentation, and AR modeling of the renal cancer with the artery, vein, and collecting system

A 56-year-old male presented with right upper quadrant/flank pain. Imaging was performed on a 1.5 T MR System (Avanto, Siemens, Erlangen, Germany). A 3D post-contrast fat-suppressed gradient-echo T1-weighted sequence with an interpolated spatial resolution of 1.4 mm × 1.4 mm × 2 mm was acquired. Standard sequence parameters were: TR = 3.58 ms, TE = 1.3 ms, FA = 12°. Imaging demonstrated a 4.4 × 4.0 cm enhancing right renal lesion (nephrometry score = 9a) that contacted the right upper and mid pole calyces. The kidney, lesion, artery, vein, and collecting system were segmented and post-processing was performed as described above. Finally, in preparation for the surgery, an AR renal cancer model was created. Figure 9 shows the entire workflow from image acquisition to model creation for this patient. At surgery, there was complete excision of the mass with gross negative margin; warm ischemia time (WIT) was 35 min; ~ 80% of normal renal parenchyma was spared.

### Prostate Cancer

#### Case 1

**Fig. 10:**
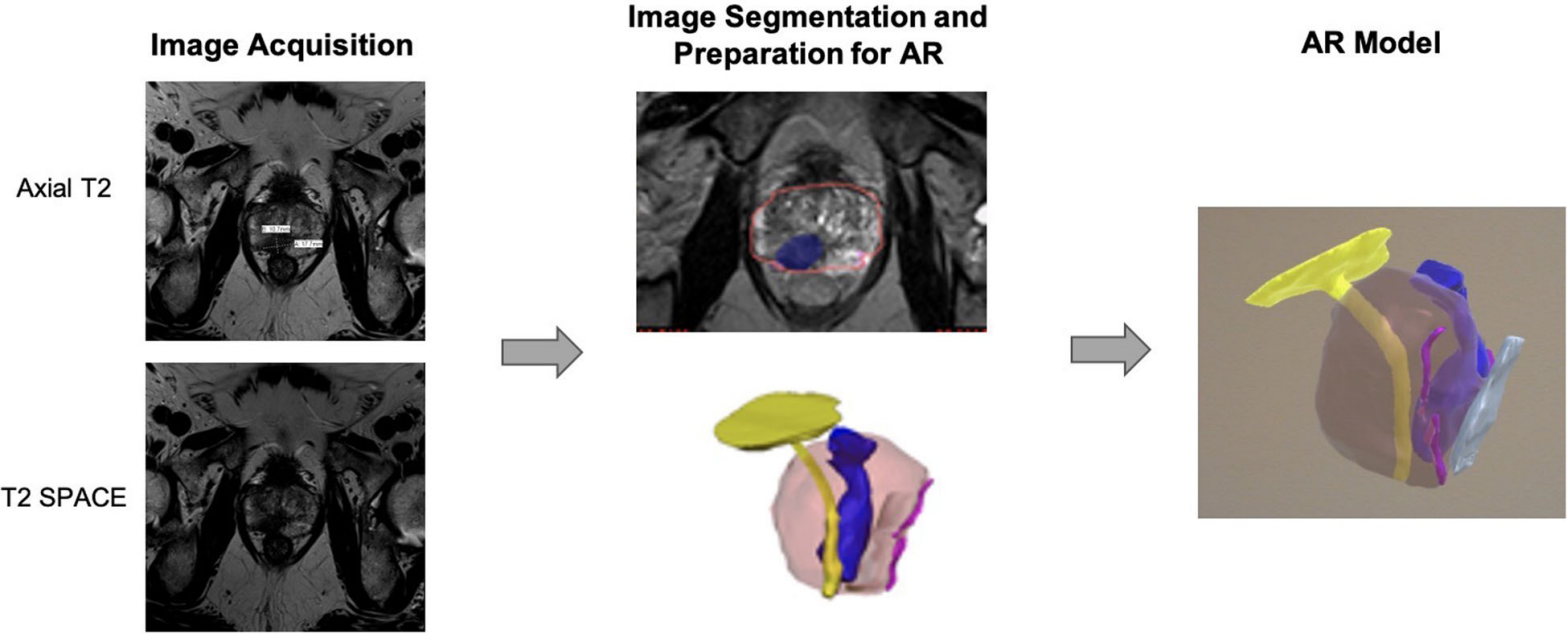
Image Acquisition**,** segmentation, and AR modeling of the prostate cancer with the lesion (blue), bladder neck and urethra (yellow), neurovascular bundles (pink), and rectal wall (white)

A 66-year-old male initially presented with an elevated PSA of 6.0 ng/ml. Multi-parametric 3 T pelvic phased-array coil prostate MRI (Siemens, Erlangen, Germany), including multi-planar T2WI, 3D T2WI SPACE, DWI (with a calculated b-1500 image set), and DCE images were performed. Prostate MRI demonstrated an 18 × 11 mm right peripheral zone lesion assigned a PI-RADS assessment score of 5, with associated extra-prostatic extension. MRI-ultrasound fusion targeted biopsy demonstrated Gleason score 3 + 4 tumor. The patient decided to undergo prostatectomy. Image segmentation of the prostate, lesion, urethra and bladder neck, neurovascular bundles, and rectal wall was performed using the T2WI SPACE sequence. Image post processing was completed in 3-matic and the AR model was built in the Unity software. In this case, the model helped the patient to better understand the size of his cancer and the surgeon to fully appreciate the extent of extra-prostatic extension (EPE) **(**Fig. 10**)**.

#### Case 2

**Fig. 11:**
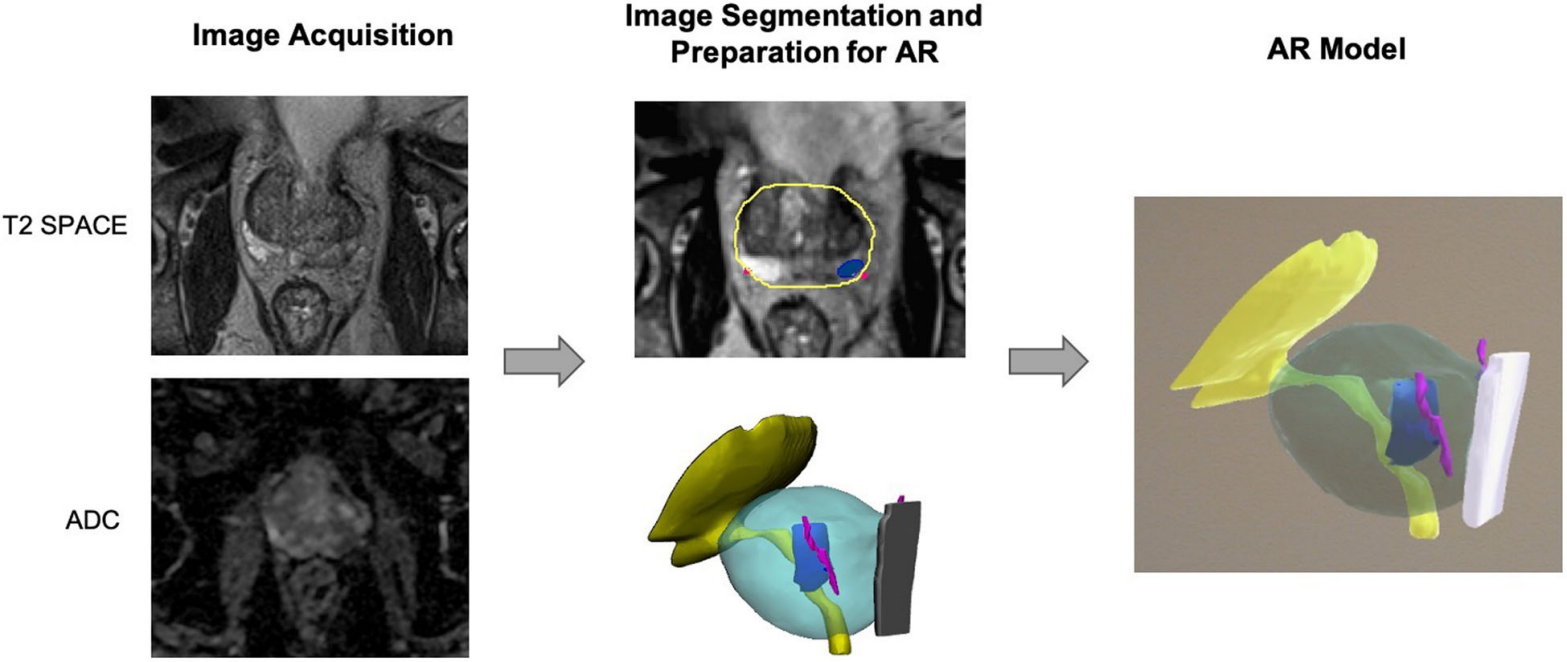
Image Acquisition**,** segmentation, and AR modeling of the prostate cancer with the lesion (blue), bladder neck and urethra (yellow), neurovascular bundles (pink), and rectal wall (white)

A 74-year-old man presented with a PSA of 5.3 ng/ml. Prostate MRI was performed as above; and it demonstrated a 14 × 10 mm posteromedial midgland-to-apex peripheral zone lesion that was assigned a PI-RADS score of 4. MRI-ultrasound fusion targeted biopsy demonstrated Gleason score 4 + 3 tumor. The patient elected to undergo radical prostatectomy. In order to create the AR model, image segmentation and post-processing were performed. The lesion was manually segmented on the DWI images and the remaining structures were segmented on the SPACE images. The segmented anatomy was co-registered using a 3D rigid registration method consisting of translation and rotation. 3D meshes were generated and the contours were generated onto the original source images to verify accuracy. The AR model was created in the Unity software and was used to plan the procedure **(**Fig. 11**)**. In addition, visualizing the AR model helped the patient to understand why the nerve could not be preserved in this case.

## Conclusions

A method for viewing patient specific cancer models in AR has been successfully implemented and described. Visualizing complex medical imaging data as real 3D anatomical models in AR can facilitate understanding of complex surgical anatomy by helping to depict the relationship of dominant tumors to key anatomic structures, allow surgeons to prepare for complex surgical cases, and enable patients to better understand their disease and treatment plan.

We have previously shown that pre-operative 3D printed models of renal mass are valuable tools that facilitate pre-operative planning by allowing surgeons to assess both tumor complexity and the relationship of the tumor to major anatomic structures such as the renal vasculature and collecting system [26, 27]. 3D printed and AR models can also allow patients to better comprehend their disease and the surgical procedure [28]. Herein, the AR cancer models also provided surgeons with an improved understanding of the tumor anatomy and can be useful during the operative procedure.

As compared to VR or 3D printed models, AR models can be co-registered to the surgical anatomy during the surgical procedure providing a real-time road map for the surgical team. A potential future application will be to have AR technologies integrated into the operating room systems, enabling surgeons to perform patient-specific surgical rehearsals and providing real-time surgical navigation. To date, several studies have described methods of using AR models that are rendered directly in the DaVinci robotic console (Intuitive Surgical, Sunnyvale, CA) to provide real-time surgical guidance [29–31]. It is expected that more work on this topic will be performed and intra-operative navigation will be extended to other AR platforms. In addition, it is expected that future systems which utilize co-registration methods will account for body motion or changes in patient positioning.

Current barriers to AR at this time include the long image segmentation and post-processing times as well as the absence of a Current Procedural Terminology (CPT) code that would provide a reimbursement method beyond patient billing, reliance on institutional funding, or research grant funding. In 2019, four category III CPT codes were released by the American Medical Association (AMA) for 3D printed anatomic models and guides [32]. Although these category III codes are for new technologies and are limited in the amount of reimbursement that may be obtained, the establishment of these codes will allow for an application for category I CPT codes, which are fully reimbursable, in the future. If there is evidence of widespread use of AR in medicine and the clinical value of using AR has been demonstrated, it is possible that AR modeling will follow a similar path as 3D printed models.

Another important factor is the accuracy of the models and co-registration of these models if they’re being used for real time surgical navigation. For co-registration of models used for incision planning and intra-operative guidance, it is imperative that patient motion and deformation are accounted for. A systematic quality assurance approach should be established to ensure the accuracy of each step during the AR process including image acquisition, segmentation and processing, and co-registration of AR content. It is expected that Quality Assurance (QA) programs for AR models will be similar to those that are being established for 3D printed anatomic models and guides [33, 34].

Improvements in AR, VR, and 3D printing technologies are continuously being made and an increasing number of groups are using these technologies in their medical practices. Each method of advanced visualization method has its advantages with 3D printing providing tactile feedback, VR allowing for a fully immersive experience, and AR enabling real-time surgical navigation. In the future, it is expected that surgeons will be able to request 3D models to enhance conventional medical image visualization and that the model type will depend on the procedure type, time frame, surgeon preference, and available utilities. As more institutions implement these technologies in the clinic, radiologists and other clinicians are expected to become increasingly involved in these emerging technologies. Future studies will need to be performed to evaluate the added value of 3D printing, AR, and VR over conventional 2D image visualization.

## Data Availability

The datasets used and/or analyzed during the current study are available from the corresponding author on reasonable request.
